# Thickness Determination and Control in Protein-Based Biomaterial Thin Films

**DOI:** 10.1021/acsabm.4c00803

**Published:** 2024-07-15

**Authors:** Lisa Almonte, Maxence Fernandez, Juan David Cortés-Ossa, Paolo Blesio, Lucía Juan-Bordera, Carlos Sabater, Aitziber L. Cortajarena, M. Reyes Calvo

**Affiliations:** † Departamento de Física Aplicada, Universidad de Alicante, 03690 Alicante, Spain; ‡ Instituto Universitario de Materiales de Alicante (IUMA), Universidad de Alicante, 03690 Alicante, Spain; § Centre for Cooperative Research in Biomaterials (CIC biomaGUNE), Basque Research and Technology Alliance (BRTA), 20014 Donostia-San Sebastián, Spain; ∥ Ikerbasque, Basque Foundation for Science, Plaza Euskadi 5, 48009 Bilbao, Spain; ⊥ BCMaterials, Basque Center for Materials, Applications and Nanostructures, 48940 Leioa, Spain

**Keywords:** protein-based biomaterials, consensus tetracopeptide repeat protein, film thickness, microreflectance spectroscopy, apparent color, self-assembly, bioelectronics

## Abstract

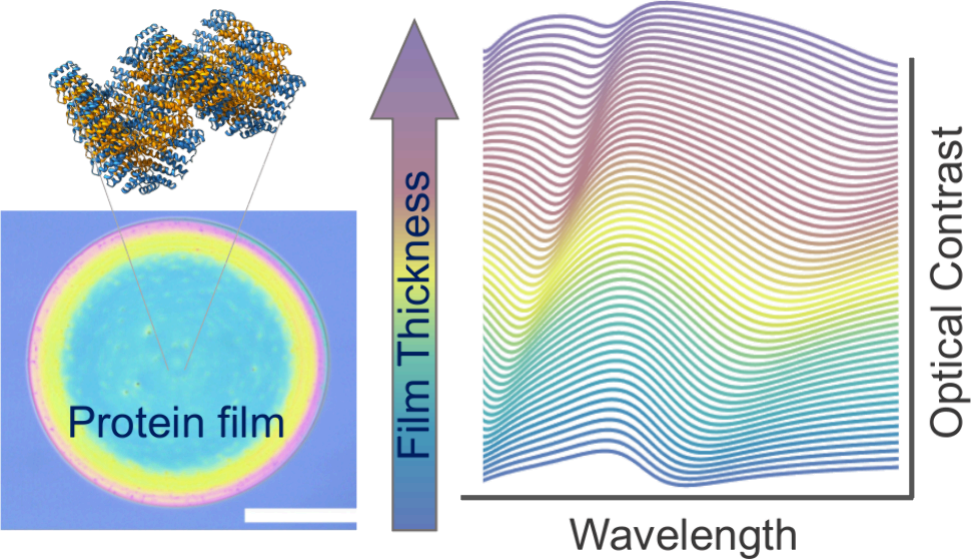

Controlling the thickness and uniformity of biomaterial films is crucial for their application in various fields including sensing and bioelectronics. In this work, we investigated film assemblies of an engineered repeat protein—specifically, the consensus tetratricopeptide repeat (CTPR) protein —a system with unique robustness and tunability. We propose the use of microreflectance spectroscopy and apparent color inspection for the quick assessment of the thickness and uniformity of protein-based biomaterial films deposited on oxidized silicon substrates. Initially, we characterized the thickness of large, uniform, spin-coated protein films and compared the values obtained from microreflectance spectroscopy with those obtained from other typical methods, such as ellipsometry and atomic force microscopy. The excellent agreement between the results obtained from the different techniques validates the effectiveness of microreflectance as a fast, noninvasive, and affordable technique for determining the thickness of biomaterial films. Subsequently, we applied microreflectance spectroscopy to determine the thickness of drop-casted CTPR-based films prepared from small protein solution volumes, which present a smaller surface area and are less uniform compared to spin-coated samples. Additionally, we demonstrate the utility of apparent color inspection as a tool for assessing film uniformity. Finally, based on these results, we provide a calibration of film thickness as a function of the protein length and concentration for both spin-coated and drop-casted films, serving as a guide for the preparation of CTPR films with a specific thickness. Our results demonstrate the remarkable reproducibility of the CTPR film assembly, enabling the simple preparation of biomaterial films with precise thickness.

## Introduction

In recent years, protein-based biomaterials have attracted significant interest due to their inherent biocompatibility and their potential applications in a range of fields, including drug delivery, tissue engineering, biosensing, or bioelectronics.^1−8^ Beyond biocompatibility, many of these applications require robust protein assemblies, and precise control over their structural, mechanical, and functional properties.^9^ Through the past 20 years, various processes have been developed to implement protein-based biomaterials into hydrogel, foam, particle, fiber, or film assemblies suitable for real-life applications.^10−13^ Although proteins can maintain their structural features through processing, the final performance of the processed material highly depends on thorough control over the specific properties of the resulting assemblies. In particular, biomaterial films have great potential for applications in sensing and bioelectronics. For example, film homogeneity is key for recognition and reproducibility in sensing applications.^14^ Importantly, film thickness and uniformity are crucial for electrical conduction phenomena and thus condition the use of biomaterials as active bioelectronic materials.^1,15,16^

Within protein-based biomaterials, assemblies from engineered proteins offer functional and structural advantages for a range of applications. Thanks to their modular and hierarchical structures, repeat proteins emerge as an effective tool for exploring the potential of multilayered and self-assembled bottom-up biomaterials. Repeat proteins are made up of a repetitive arrangement of a single structural module, such as the tetratricopeptide repeat (TPR).^17,18^ Increasing the number of TPR units within a protein results in a rigid, right-handed superhelical structure.^19,20^ Moreover, a combination of head-to-tail stacking and weak dipole–dipole interactions drive the supramolecular self-assembly of CTPR proteins,^21,22^ allowing the preparation of thin CTPR films through spin-coating or drop-casting of protein solutions.^22^ CTPR assemblies can be tailored to exhibit various physical and chemical properties. This, coupled with their unique structural characteristics, make CTPRs a promising molecular unit to generate tailored biomaterials with significant potential for applications in bio-optical and bioelectronic devices.^23−26^

Precise control of the thickness of protein-based films is essential for various applications, especially toward their implementation in microdevices. Among the most common techniques used to characterize the thickness of organic thin films are optical transmittance and reflectance,^27−30^ ellipsometry,^31−34^ profilometry,^35,29^ and atomic force microscopy (AFM).^36−39^ Transmittance and reflectance methods, which involve analyzing the intensity of light that passes through or is reflected from the sample, respectively, as a function of wavelength, provide an accurate determination of the thickness of organic films.^40−43^ Ellipsometry is a nondestructive optical technique that measures differences in amplitude and phase between the parallel and perpendicular components of a polarized light beam after reflection from a surface.^44,45^ Upon use, ellipsometry provides comprehensive information about the thickness and refractive index of samples.^46^ In biotechnology, spectroscopic ellipsometry has been routinely applied to assess the surface coverage and thickness of protein films on various substrates and electrodes.^47−51^ Profilometry measures the height profile and roughness of samples by tracing a stylus or probe along their surface. Similarly, in AFM, a nanoscale tip probes the surface topography of samples with nanometer resolution. AFM has been widely applied to the local characterization of the thickness and roughness of organic films^52^ and biomaterial assemblies.^53−56^ Finally, other techniques, such as surface plasmon resonance (SPR) measurements have also been applied to quantify the thickness of biomaterial films.^57^

Even if widely applied, each of the techniques described above has its limitations. On the one hand, typical optical transmittance, reflectance, and ellipsometry spectroscopies rely on the assumption of uniformity and extensiveness of the film, which can lead to inaccuracies when the assumption is not valid. Also, transmittance measurements are restricted to films deposited on transparent substrates. In the case of ellipsometry, comprehensive characterization comes at the expense of sophisticated instrumentation and data analysis. On the other hand, profilometry and AFM are suitable for local thickness measurements in relatively small-area or nonuniform samples. However, they can be time-consuming and invasive methods, since measuring film thickness with these techniques often involves scratching the samples to create a step between the film and the exposed substrate.^35,36^ Finally, other noninvasive techniques, such as SPR, are suitable for nonuniform small-area films, but their use is restricted to samples on metallic substrates and requires specialized experimental setups. In contrast, microtransmittance and microreflectance spectroscopy techniques offer a fast, accurate, noninvasive method to characterize the thickness and uniformity of thin films, even for nonuniform films or samples with small surface areas.^58−60^ These techniques provide spatially resolved information with micrometer resolution and require relatively simple, nonsophisticated instrumentation, and data analysis. Specifically, microreflectance is suitable for use with samples on transparent and nontransparent substrates and has been widely applied for the thickness determination of different inorganic and organic thin films.^61^

In this work, we propose the use of microreflectance spectroscopy for a fast and noninvasive thickness determination of biomaterial assemblies, focusing specifically on CTPR protein-based films. We demonstrate the efficacy of this technique, particularly emphasizing its utility for nonuniform films and samples with small surface areas. First, to corroborate the validity of the microreflectance spectroscopy method, we compared the thickness values obtained by this technique with those estimated using standard thin film techniques such as AFM or ellipsometry. This comparative analysis was conducted on large, uniform CTPR films prepared by spin-coating on Si/SiO_2_ substrates, revealing good agreement between the results obtained by the different techniques. Additionally, the constructive interference of visible light on the Si/SiO_2_ substrates leads to strong changes in apparent color with varying thicknesses of the CTPR film. This feature enables a rough estimation of CTPR film thickness through simple optical inspection. Subsequently, we applied microreflectance spectroscopy and apparent color identification methods to determine the thickness of drop-casted films prepared from small volumes of CTPR solution. Based on our results, we generated a calibration guide for average CTPR film thickness as a function of protein length and concentration. Our findings will facilitate the rapid and nondestructive characterization of drop-casted protein-based biomaterial films. Moreover, the remarkable reproducibility of the CTPR system allows for the straightforward fabrication of biomaterial films with a desired thickness.

## Results and Discussion

**Figure 1:**
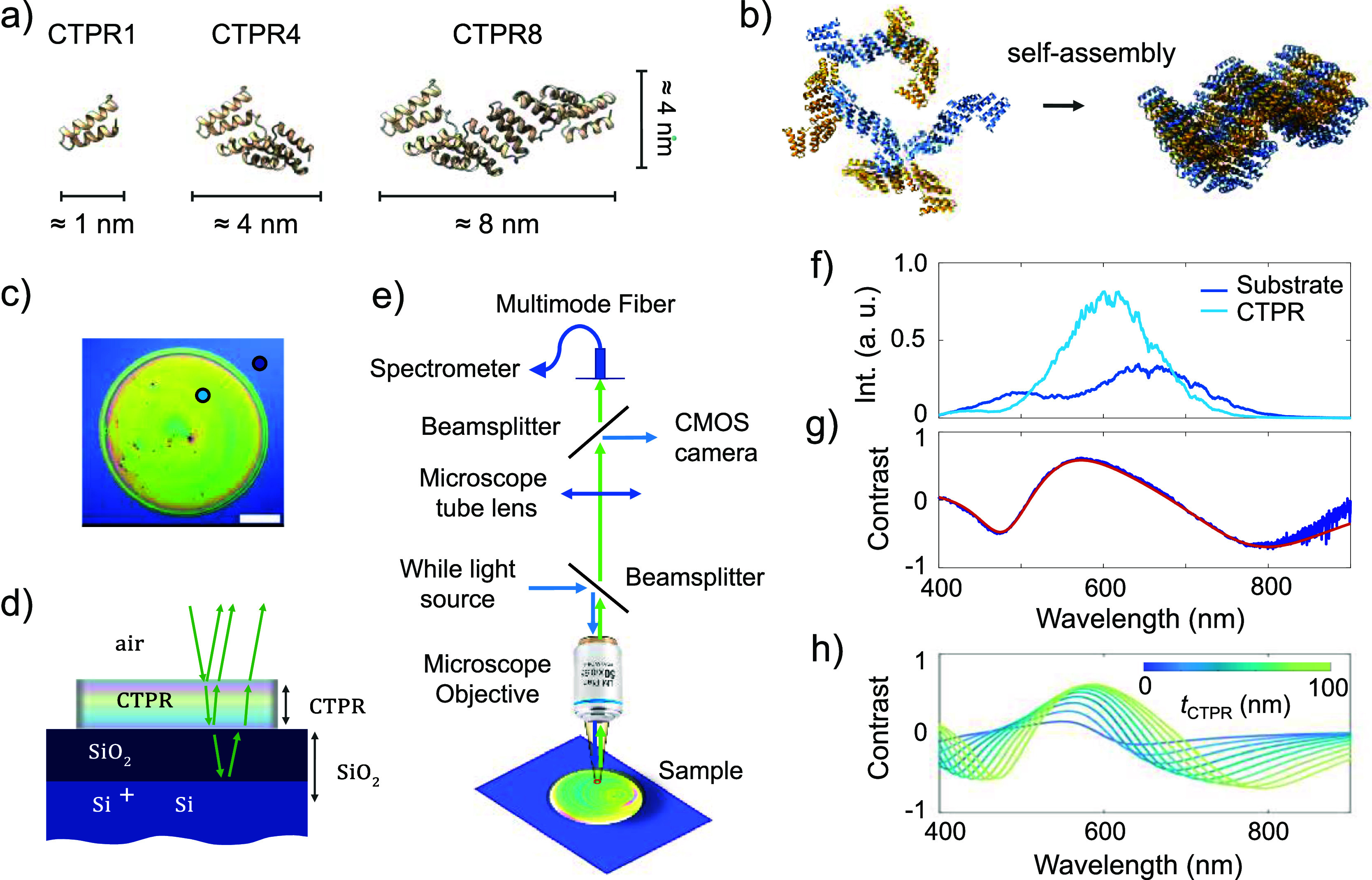
(a) Illustration of a CTPR protein with 1, 4, and 8 identical repeats (Structures based on CTPR8 crystal structure PDB ID: 2AVP(20)). (b) Sketch illustrating CTPR proteins in solution (left) and forming a highly ordered CTPR film (right) upon solvent evaporation and self-assembly. (c) Optical image of a protein-based biomaterial film prepared by drop-casting of CTPR4 solution (scale bar represents 0.5 mm). (d) Representation of incident light propagation in the multilayer stack Si/SiO_2_/CTPR. The reflected light depends on the specific thickness of the oxide layer *t*_SiO2_ and the protein film *t*_CTPR_ as well as on the refractive index of each layer (*n*_*x*_) and surrounding media (air and Si). (e) Schematics of the microreflectance spectroscopy setup. (f) Microreflectance spectra acquired over the CTPR4 film (light blue) and the bare Si/SiO_2_ substrate (dark blue) from spots of ∼3 μm diameter at the locations marked in panel c. (g) Experimental optical contrast calculated from data in panel f as indicated in the main text. The red line represents the fit of the data to a Fresnel model. The fit yielded a thickness of ∼115 nm for the CTPR4 thin film. (h) Optical contrast simulation for the multilayer stack depicted in panel d, for fixed *t*_SiO2_ = 295 nm and varying values of *t*_CTPR_ ranging from 10 to 100 nm.

The engineered CTPR unit is a 34-amino acid sequence that encodes a helix-turn-helix fold. This sequence can be repeated in tandem to generate CTPR proteins (CTPR*n*), where *n* represents the number of identical CTPR units, ranging from 2 to 20^23,24^ (Figure 1a). When spin- or drop-casted on a surface, CTPR proteins self-assemble upon solvent evaporation and result in the formation of free-standing films^22,21^ (see sketch in Figure 1b). Protein-based biomaterial film assemblies can be prepared by either spin-coating or drop-casting CTPR proteins in solution onto Si/SiO_2_ substrates. For instance, Figure 1c shows an optical image of a film formed after drop-casting 0.4 μL of a 200 μM solution of CTPR4 — a CTPR protein with 4 identical repeats — on a Si/SiO_2_ substrate with a 295 nm thick SiO_2_ layer. SEM images showing the uniformity of the surface and cross-section of the films are presented in Supporting Information S1. Si/SiO_2_ substrates are widely used for prototyping field-effect transistors, with the SiO_2_ acting as a dielectric layer for electrostatic gating. These substrates are suitable for the characterization of the electronic properties of protein-based films toward bioelectronic applications. Additionally, the CTPR film and the Si/SiO_2_ substrate form a multilayer system where the constructive interference of light (see sketch in Figure 1d) in the visible wavelength range leads to pronounced color changes with small variations in the CTPR film thickness. Later in the manuscript, we will demonstrate that the different apparent colors in the CTPR film on Si/SiO_2_ (see Figure 1c) correspond to regions with different film thicknesses.^62,60,63^ Using microreflectance spectroscopy, changes in apparent color can be quantified, and more precise thickness values can be extracted, provided the material refractive index is known.^27,60^

Figure 1e shows the schematics of a microreflectance setup, based on a modified commercial optical microscope, such as the one described in the work by Frisenda et al.^60^ In this setup, the sample is illuminated with white light from a halogen lamp, and the reflected light from the surface is collected in an optical fiber and brought to a compact spectrometer. The light is collected from a spot on the sample, and the size of this spot is proportional to both the fiber core diameter and the magnification of the microscope (see Materials and Methods).^60^ Using this setup, microreflectance spectra were acquired for locations both on the bare substrate (dark blue curve in Figure 1f) and on the region covered by the CTPR film (light blue curve in Figure 1f). Despite the spectral distribution of the intensity of the light source, clear differences can be seen in the CTPR spectrum compared to that of the bare Si/SiO_2_ substrate. To remove the influence of the spectral shape of the light source, we normalized the two spectra, as shown in Figure 1g. The optical contrast *C* is calculated as *C* = (*I*_CTPR_ – *I*_subst_)/(*I*_CTPR_ + *I*_subst_), where *I*_CTPR_ and *I*_subst_ are the microreflectance light intensity measured on the CTPR film and on the bare Si/SiO_2_ substrate, respectively (Figure 1g).

Using the refractive index dispersion for the CTPR protein films reported in ref (25) and the refractive index data for Si and SiO_2_ from ref (64), optical contrast can be simulated using a transfer-matrix method derived from Fresnel equations for light propagation in multilayer thin film stacks^65,66^ (see sketch in Figure 1d, Methods, and Supporting Information S2). The simulation in Figure 1h illustrates the evolution of optical contrast spectra for different thicknesses of the CTPR film with a fixed thickness of 295 nm for the SiO_2_ layer. In the Supporting Information S3, we present similar simulations for a silicon substrate with a native oxide layer of 5 nm. In this case, the changes in optical contrast with CTPR thickness are negligible. However, significant optical contrast changes can be observed for a ∼295 nm SiO_2_ layer and CTPR films ranging from 10 to 100 nm thick (Figure 1h). The proposed model for simulation is also used to fit experimental optical contrast data, yielding an estimation of the thickness of the CTPR4 film. In the case of the contrast data presented in Figure 1g, the thickness is determined to be ∼115 nm.

**Figure 2:**
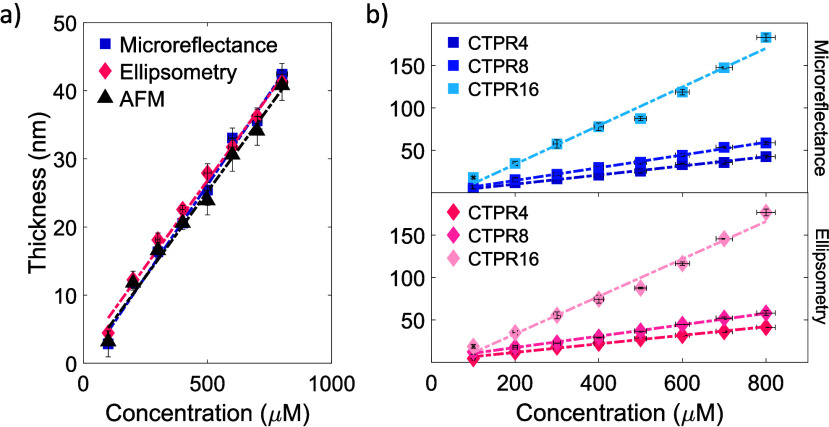
(a) Thickness as a function of concentration for CTPR4 films prepared by spin-coating corresponding protein solutions, measured using microreflectance spectroscopy, ellipsometry, and AFM topography, respectively. (b) Film thickness as a function of CTPR protein length and concentration obtained using microreflectance (top) and ellipsometry (bottom) techniques. Dashed lines represent linear fits of the data. Fitting parameters and coefficients of determination are summarized in Table S1.

To benchmark the results obtained from microreflectance analysis, we compared the thickness values obtained from microreflectance with those obtained from two other standard thin-film characterization techniques: ellipsometry and AFM. For this purpose, different CTPR solutions were spin-coated onto a Si/SiO_2_ substrate (see Materials and Methods). Spin-coated CTPR films typically cover large surface areas and present relatively uniform thicknesses compared to those prepared by drop casting. This makes them suitable for characterization by all three techniques, facilitating a comparison of results. CTPR4 (a CTPR protein with 4 identical repeats) films were prepared by spin-coating protein solutions ranging from 100 to 800 μM concentration, to produce samples with different thicknesses (see Figure S5). Three separate samples were prepared for each CTPR concentration. Microreflectance spectroscopy measurements were performed at five different locations for each sample. For each of those measurements, the thickness of the CTPR film was extracted from the fitting of the optical contrast data, as explained before. The same samples were also characterized by spectroscopic ellipsometry. In this case, a spectrum was captured for each of the CTPR films and fitted to a Cauchy model to extract their thickness (see Supporting Information S5 and Methods). Finally, AFM in noncontact amplitude modulation mode was used to determine the height of the same protein films. Prior to measurement, a gentle scratch was performed on the sample using a pipette tip. Care was taken to avoid any damage to the silicon oxide layer as it could impact the accuracy of height measurements (see Materials and Methods and Supporting Information S6). This process was repeated at three different locations for each sample. The main thickness values and corresponding error, presented in Figure 2a and Table 1, were derived from the mean and standard deviation of all measurements performed by each technique on films prepared from the same protein concentration (Figure 2a, Table 1).

**Table 1:** Thickness Characterization of Films Prepared by Spin-Coating CTPR4 Solutions at Eight Different Concentrations^a^

CTPR4 concentration (μM)	microreflectance thickness (nm)	ellipsometry thickness (nm)	AFM topography thickness (nm)
100 ± 3	3 ± 2	5 ± 1	3 ± 1
200 ± 6	12 ± 2	12 ± 1	12 ± 1
300 ± 8	16 ± 2	18 ± 1	17 ± 1
400 ± 10	21 ± 2	23 ± 1	21 ± 1
500 ± 10	25 ± 2	28 ± 1	24 ± 2
600 ± 20	33 ± 2	32 ± 1	31 ± 2
700 ± 20	36 ± 2	36 ± 1	34 ± 2
800 ± 20	42 ± 2	41 ± 1	41 ± 2

a Thickness values are obtained from the average of multiple measurements, and error values are calculated from the standard deviation. Ellipsometry analysis was performed for each of the three separate samples prepared at each protein concentration. AFM measurements are performed at three different locations, while micro-reflectance measurements are taken at five different locations for each of the three samples at each concentration.

The thickness values obtained from the three techniques are consistent within the margin of error (Figure 2a, Table 1), corroborating the validity of the microreflectance analysis, which provides film thickness with a precision of 2 nm. Even if the three techniques yield similar results, they present slight differences. Specifically, both AFM and microreflectance derive thickness from local measurements, whereas ellipsometry provides an averaged thickness from a few square millimeters area of the sample. By comparing data obtained at different spots on the sample, both microreflectance and AFM offer the advantage of assessing the thickness uniformity of the sample and the capability to analyze specific areas of interest with spatial resolution. Remarkably, compared to AFM, microreflectance holds a significant advantage due to its nondestructive and fast data acquisition capabilities.

The results obtained from all three techniques consistently indicate that the film thickness increases linearly with protein concentration (Figure 2a). Various other factors, such as molecular weight and conformation, may contribute to variations in film thickness. Specifically, exploring the influence of the length of the CTPR protein on the film thickness may provide valuable knowledge about the fundamental aspects of film formation. To investigate the relationship between film thicknesses and CTPR protein lengths, a set of samples of CTPRs with different numbers of repeats was prepared by spin coating. The thicknesses of films prepared from varying concentrations of CTPR proteins with 4, 8, and 16 repeats are presented in Figure 2b and summarized in Tables S2 and S3. Here too, the thickness values obtained from microreflectance spectroscopy and ellipsometry measurements show good agreement (see Figure 2b). For all protein lengths, adjusting the concentration of the CTPR protein has a significant influence on film thickness, with higher concentrations of protein solutions resulting in thicker films. The slope obtained from the linear fit of thickness-concentration data increases with the protein length (see Figure 2b and Table 2, other fit parameters are presented in Table S1). These results provide a practical guide for the preparation of CTPR films with a specific thickness for a given protein length. Thickness values for the three protein lengths, as a function of mass concentration (mg/mL), overlap in the lower concentration range (see Supporting Information S9), suggesting that the resulting film thickness is mainly determined by the total amount of protein deposited. For higher mass concentrations, such as those used for CTPR16, the thickness-concentration slope increases (see Figures 2b, S9, and S10), resembling the behavior of spin-coated films prepared with standard polymers such as polystyrene.^67^

**Table 2:** Calibration of CTPR Film Thickness as a Function of Protein Concentration and Length, Represented by the Slopes Obtained from the Linear Fit of Thickness-Concentration Data Presented in Figures 2b and 4b, for Spin-Coated and Drop-Casted Samples, Respectively^a^

protein	spin-coated films thickness-concentration slope (nm/μM)	drop-casted films thickness-concentration slope (nm/μM)
CTPR4	0.054 ± 0.005	0.50 ± 0.03
CTPR8	0.075 ± 0.004	1.2 ± 0.1
CTPR16	0.23 ± 0.03	3.0 ± 0.3

a The error values are estimated from the 95% confidence intervals of the linear fit.

**Figure 3:**
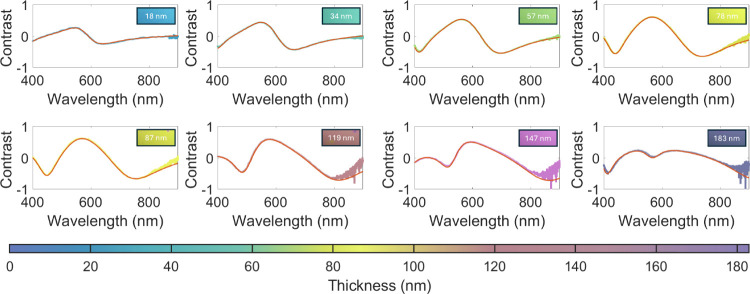
Optical contrast spectra obtained for CTPR4 films of different thickness. Red lines represent fits to a transfer matrix model based on the Fresnel equations (see Supporting Information S2). The insets present bright-field optical microscopy images corresponding to the film areas in which spectra were taken.

**Figure 4:**
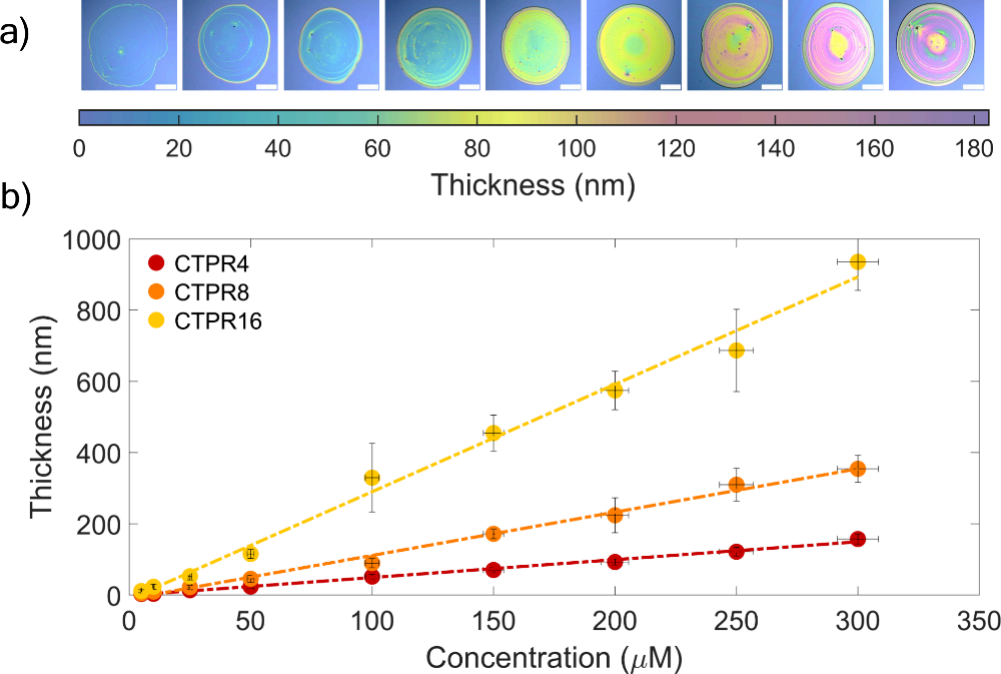
(a) Optical images of drop-casted CTPR4 films prepared from solutions with increasing protein concentration. The scale bar represents 1 mm. (b) Thickness as a function of protein length and concentration for films prepared by drop-casting. Thickness is extracted from the average of five different microreflectance measurements performed in the central area of three separate films prepared at each concentration. Error is obtained from the standard deviation of results. Dashed lines represent linear fits to the data. Fitting parameters and coefficients of determination are summarized in Table S1.

Changes in the optical contrast, as measured by microreflectance spectroscopy, enable a quantitative assessment of protein film thickness with microscale spatial resolution. Nonetheless, variations in optical contrast corresponding to different film thicknesses are also discernible in the optical images of the samples; areas with different thicknesses appear with distinct apparent colors (Figure 3). Although the precise correlation between color and thickness depends on the specifics of the optics and the camera CMOS sensor, a color guide can be constructed by associating apparent colors with film thickness values obtained from the analysis of optical contrast spectroscopy (Figure 3). This color guide, presented at the bottom of Figure 3, serves as a practical tool for a quick estimate of CTPR film thickness within a range between 10 and 200 nm, with ∼20 nm error. This method offers a convenient alternative to specialized equipment, relying solely on visual inspection. Moreover, the variations in apparent color can also serve as indicators of film homogeneity and thickness uniformity. For example, apparent color inspection aids in qualitatively identifying thickness variations between different regions of the film. This is particularly useful for determining the thickness uniformity of drop-casted films, such as the ones presented in Figure 4a, where different colors can be observed from the film’s edges to its center.

Next, we apply microreflectance spectroscopy to determine the thickness of drop-casted CTPR films prepared out of small volumes of material. Figure 4a shows drop-casted films prepared out of 0.4 μL of solutions with different CTPR4 concentrations (5–300 μM). The films prepared using this method cover relatively smaller areas (∼3–5 mm^2^) and can be of interest for microdevice preparation and electrical characterization. The apparent color inspection of drop-casted films suggests that these films are less uniform than those prepared by spin-coating, with the formation of relatively homogeneous thinner areas in the center of the film and thicker areas toward the edge (Figure 4a). These samples are unsuitable for typical ellipsometry characterization, making the local thickness determination provided by microreflectance spectroscopy particularly advantageous in such cases. To determine the average thickness of the more uniform central areas of drop-casted CTPR films, microreflectance measurements were performed at five different locations within that region for each sample. The process was repeated for three separate samples prepared at each concentration. Subsequently, thickness values and corresponding errors were derived from the mean and standard deviation of the five measurements, respectively. The resulting thickness values are presented in Figure 4b and are summarized in Table S4. Similar to spin-coating samples, the thickness of drop-casted films presents a nearly linear correlation with the protein concentration. Like the spin-coating data, the slope of the linear fit to the thickness-concentration data increases with the increasing protein length (see Table 2). A comparison of the results obtained using spin-coating and drop-casting methods for film preparation reveals that the drop-casting technique consistently produces thicker films than spin-coating for the same CTPR concentration (Table 2). The linear thickness dependence on protein concentration for different molecular lengths suggests high reproducibility in the film assembly for the CTPR system. In the case of drop-casted samples, doubling the protein length results in a slightly more than twice increase in the thickness-concentration slope. This can be also observed in the slightly larger slopes of thickness-mass concentration data for longer variants (see Supporting Information S9). These results suggest that while protein quantity appears to dominate the resulting thickness of the assembly, other factors might introduce variations that seem to depend on protein length and preparation method. The obtained thickness-concentration ratios in Table 2 can be used as a calibration guide to prepare films of specific thickness.

**Figure 5:**
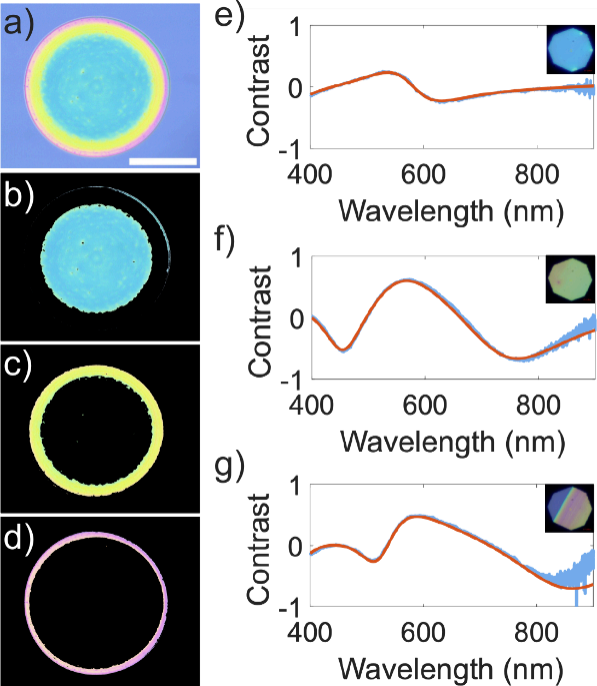
(a) Microscope image of a CTPR8 film deposited by drop-casting 0.4 μL of solution at 25 μM. The scale bar represents 1 mm. (b–d) Segmentation of the film image in panel a into three different apparent color regions derived from the application of a k-means clustering method. (e–g) Optical contrast spectra acquired at representative spots of each of the three segmented regions in panels b–d, respectively. Insets show optical images of the areas where spectroscopy was performed. Red lines represent fits of the contrast data to the model described in the Supporting Information S2. Thickness values are extracted from these fits.

Finally, we employed a k-means clustering method for image segmentation (see Materials and Methods) to identify film regions with different thicknesses. The film image presented in Figure 5a, following segmentation, was cropped into three distinct apparent color regions (Figure 5b–d), corresponding to three main thickness values. The average thickness of each region was estimated from microreflectance spectroscopy measurements (Figure 5e–g, Table 3). Using these values and the estimated areas for the different thickness regions, we can estimate the volume of the film (see Table 3).

**Table 3:** Area and Thickness Values Estimated from the Segmented Images in Figure 5b–d and Optical Contrast Spectra in Figure 5e–g, Respectively^a^

cluster	area (mm^2^)	thickness (nm)	volume (10^3^ μm^3^)
(b)	2.0	17	34
(c)	0.9	96	84
(d)	0.5	151	72
total	3.4		191

a From these values, the film volume associated with each segment and the total volume of the film are estimated.

## Conclusions

On the one hand, this work demonstrates the efficacy of microreflectance spectroscopy and apparent color identification methods as rapid, noninvasive, and affordable techniques for determining the thickness of protein-based biomaterial thin films. Specifically, we successfully applied these methods to estimate the thickness of drop-casted films prepared from small volumes of CTPR protein solutions. These films present small surface areas and nonuniform thickness distributions, making thickness measurements by other standard techniques, such as ellipsometry, inaccurate. Through the application of microreflectance spectroscopy, we can track spatial variations of film thickness, assess the uniformity of film thickness, and provide accurate thickness values for relatively uniform regions of the films. In summary, this characterization can contribute to a better understanding of film formation processes and guide the optimization of protein-based and other biomaterial film deposition for various applications. Additionally, we provide an apparent color guide for a fast qualitative assessment of the thickness of films deposited on oxidized silicon substrates. This can be particularly useful for bioelectronic devices, where oxidized silicon substrates can be advantageous.

On the other hand, the thickness characterization performed in this work serves as a calibration guide for CTPR film thickness as a function of protein concentration and length, both for spin-coated and drop-casted samples. This technical information is crucial for future research involving CTPR thin films. Furthermore, our findings contribute valuable insights into the impact of protein concentration and length on film formation, showcasing outstanding reproducibility in film assembly for CTPR systems. These properties position the CTPR platform as a strong candidate for applications where precise control of the film thickness is paramount, such as sensing and bioelectronics.

## Materials and Methods

### Sample Preparation

CTPR4, 8, and 16 were recombinantly expressed from pET-28b plasmids encoding the corresponding genes. The His-tag fusion proteins were overexpressed in *Escherichia coli* C41 cells and purified by affinity purification as described before.^68^ Protein fractions were analyzed by SDS-PAGE electrophoresis and mass spectrometry to verify their molecular mass and purity (Supporting Information S10). The protein concentration was estimated using the molar extinction coefficient at 280 nm calculated from the amino acid composition. Purified proteins were stored at −80 °C. Prior to film formation, proteins were dialyzed into Milli-Q water.

CTPR films were prepared on Si/SiO_2_ substrates with a dry thermal oxide layer of 295 nm, purchased from Si-Mat. The substrates were cleaned by sonication in acetone and isopropanol, followed by argon plasma cleaning for 30 s. CTPR films were prepared by using both spin-coating and drop-casting methods. For spin coating, 40 μL of the CTPR solution at different concentrations (100–800 μM) were carefully pipetted onto the clean Si/SiO_2_ substrates. The sample was then spun at 3000 rpm for 120 s to ensure complete drying of the film. In the case of drop-casting, a volume of 0.4 μL at different concentrations (5–300 μM) was deposited onto clean Si/SiO_2_ substrates right after plasma cleaning. Three separate samples at each concentration were prepared for both spin-coated and drop-casted films.

### Microreflectance Spectroscopy Characterization

Microreflectance measurements are performed in a modified metallurgic microscope Motic BaMET310 following the work of Frisenda et al.^60^ A halogen lamp is used for sample illumination, and reflected light from the sample is coupled to an optical fiber. The diameter of the collection spot is proportional to the diameter of the optical fiber core, which is 100 μm in our setup, leading to a collection spot size of ∼2.7 μm.

### Optical Contrast Simulations

Optical contrast *C* is calculated as the ratio of the reflectance on the CTPR/SiO_2_/Si multilayer stack (*R*_CTPR_) and the reflectance on the bare SiO_2_/Si substrate (*R*_Si/SiO2_) as *C* = (*R*_CTPR_ – *R*_Si/SiO2_)/(*R*_CTPR_ + *R*_Si/SiO2_). Reflectance values as a function of wavelength are calculated using the transfer-matrix method for light propagation in multilayer thin films^65,69^ (see Supporting Information S2).

### Atomic Force Microscopy Characterization

Atomic force microscopy (AFM) measurements on CTPR films were performed using an NTEGRA SPECTRA AFM complex from NT-MDT Spectrum Instruments Company, with AC-240 cantilevers in AFM in noncontact amplitude modulation mode. Image processing was performed by using Gwydion Software.

### Scanning Electron Microcopy

Scanning electron microscopy (SEM) measurements of the CTPR films (Supporting Information S1) were carried out with a scanning electron microscope, JSM-IT800 from JEOL. Protein samples were deposited on Si/SiO_2_ substrate using two different techniques, drop-casting, and spin-coating, and broken by freeze-fracture. SEM images were then collected with two different setups to maximize the quality of the different images. For the drop-casted sample, images were collected by an SED detector with a WD of 4.0 mm using a 1 kV beam acceleration voltage, while for the spin-coated sample, images were collected by an SED detector with a WD of 6.0 mm using a 3 kV beam acceleration voltage.

### Ellipsometry Characterization

Spectroscopic ellipsometry was performed at room temperature on a spectroscopic rotating compensator ellipsometer (M2000 V, Woollam, NE, USA). Measurements were run in a 380–1000 nm wavelength range, using an angle of incidence of 70°. To calculate the thickness of the protein films, data were processed using the Complete EASE software using a three-layer model to fit the results with the lowest mean standard deviation. The protein film was modeled as a Cauchy layer,^70^ and the silicon oxide is modeled as a layer of fixed thickness. While fitting, the calculated refractive index of the protein film was compared to an expected value (≈1.5), in agreement with previous studies (see for example ref (25)) to ensure the validity of the fits (see Supporting Information S5).

## Supporting Information Available

Optical contrast simulation details, additional data and analysis, including ellipsometry, SEM, and AFM topography data, and analysis of film thickness as a function of mass concentration (PDF)
