# Supplementary material for: Thickness Determination and Control in Protein-Based Biomaterial Thin Films
Source: ACS Appl Bio Mater. 2024 Jul 15;7(8):5719–27. doi: 10.1021/acsabm.4c00803 (PMC11337159; doi:10.1021/acsabm.4c00803)
Supplement: Supplementary file 1 — mt4c00803_si_001.pdf [file mt4c00803_si_001.pdf]

Supporting Information for

Thickness determination and control in protein-based biomaterial thin films

Lisa Almonte[1,2,*], Maxence Fernández[3], Juan David Cortés-Ossa[1,2], Paolo Blesio[3], Lucía Juan-Bordera[1,2], Carlos Sabater[1,2], Aitziber L. Cortajarena[3,4,*], M. Reyes Calvo[1,2,4,5,*]

[1] Departamento de Física Aplicada, Universidad de Alicante, Alicante 03690, Spain

[2] Instituto Universitario de Materiales de Alicante (IUMA), Universidad de Alicante, Alicante 03690, Spain

[3] Centre for Cooperative Research in Biomaterials (CIC biomaGUNE), Basque Research and Technology Alliance (BRTA), Paseo de Miramón 194, Donostia-San Sebastián, 20014 Spain

[4] IKERBASQUE, Basque Foundation for Science, Plaza Euskadi 5, 48009 Bilbao, Spain

[5] BCMaterials, Basque Center for Materials, Applications and Nanostructures, 48940 Leioa, Spain

*Email: lisa.almonte@ua.es, alcortajarena@cicbiomagune.es, reyes.calvo@ua.es

List of contents

S1. SEM characterization of CTPR films

S2. Transfer-matrix method for optical contrast simulations

S3. Contrast simulation for CTPR films on native oxide silicon substrates

S4. Optical images for spin-coated CTPR4 films at different concentrations

S5. Ellipsometry characterization of spin-coated CTPR samples

S6. Atomic force microscopy characterization of spin-coated CTPR samples

S7. Linear regression parameters

S8. Film thickness as a function of concentration for different CTPR lengths.

S9. Thickness as a function of mass concentration for spin-coated and drop-casted CTPR samples

S10. Purification and characterization of CTPR4, CTPR8 and CTPR16

Supporting references

S1. SEM characterization of CTPR films

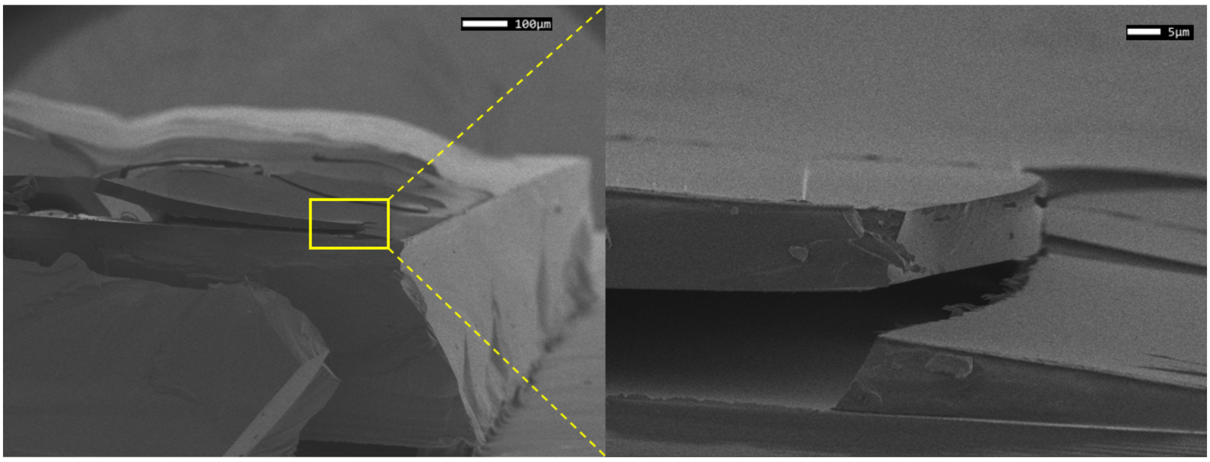

Figure S1. SEM micrograph of a cross section after freeze-fracture of a drop-casted protein film (at 800 µM CTPR4) deposited on a 525 µm Si/SiO_2 wafer with a ~295 nm layer of SiO_2. Scale bars represent 100 µm (left image) and 5 µm (right image).

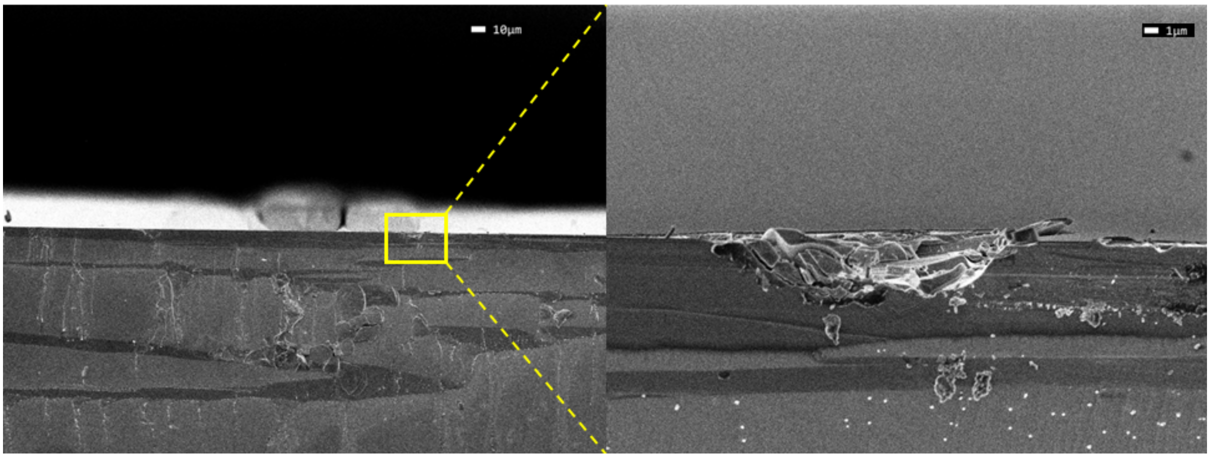

Figure S2. SEM micrograph of a cross section after freeze-fracture of a spin-coated protein film (at 800 µM CTPR4) deposited on a 525 µm Si/SiO_2 wafer with a ~295 nm SiO_2 layer. Scale bars represent 10 µm (left image) and 1 µm (right image).

S2. Transfer-matrix method for optical contrast simulations

Following the work by Byrnes *et al.* [s1], we apply the transfer-matrix method to describe the propagation of light in the multilayer system constituted by the CTPR and the SiO_2 films surrounded by two semi-infinite media, air, and Si, respectively (see sketch in Fig. S3).

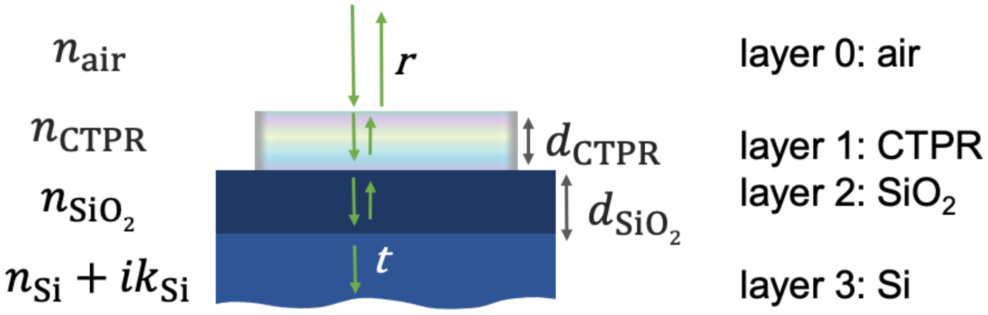

Figure S3. Schematics of light propagation in a multilayer system constituted by a semi-infinite air layer (layer 0), a CTPR thin film of thickness d_{CTPR} (layer 1), an oxide layer of thickness d_{SiO2} (layer 2) and a semi-infinite silicon substrate (layer 3). r and t represent the reflection and transmission coefficients for the total system.

The system can be described by a matrix M relating the incident, the reflected and transmitted amplitudes:

$$\begin{pmatrix} 1 \\ r \end{pmatrix} = \begin{pmatrix} M_{00} & M_{01} \\ M_{10} & M_{11} \end{pmatrix} \begin{pmatrix} t \\ 0 \end{pmatrix} \qquad (s1)$$

where r and t are the reflection and transmission coefficients for the whole system.

The matrix M can be written as the product of a series of matrices representing the reflection and transmission of light at each interface between layers and the propagation of light within each of the thin film layers.

For each interface between layers i and i+1, under normal incidence conditions, a matrix $T_{i,i+1}$ can be defined as

$$T_{i,i+1} = \frac{1}{t_{i,i+1}} \begin{pmatrix} 1 & r_{i,i+1} \\ r_{i,i+1} & 1 \end{pmatrix} \qquad (s2)$$

where the reflection ($r_{i,i+1}$) and transmission ($t_{i,i+1}$) coefficients are given by

$$r_{i,i+1} = (n_i - n_{i+1})/(n_i + n_{i+1});$$
$$t_{i,i+1} = 2\, n_i/(n_i + n_{i+1});$$

with n_i being the complex refractive index of the corresponding i-layer.

The phase changes due to propagation within each layer are considered in

$$D_i = \begin{pmatrix} e^{-i\delta_n} & 0 \\ 0 & e^{i\delta_n} \end{pmatrix} \qquad (s3)$$

where phase change δ_i of light propagating in a layer of thickness d_i is given by $\delta_i = 2\pi n_i d_i/\lambda$, with λ being the wavelength of light.

In our case, the transfer matrix for the system constituted by the four layers, sketched in Figure S3, is given by:

$$M = T_{0,1} D_1 T_{1,2} D_2 T_{2,3}$$

Given the refractive index and thickness values for each layer, the reflection coefficient r can be calculated from eq. s1 as $r = M_{10}/M_{00}$. The reflectance R of the multilayer system is then given by $R = |r|^2$.

Optical contrast simulations are performed by computing, for each wavelength value, the reflectance of the multilayer system containing the CTPR film (R_{CTPR}) and the reflectance of the bare SiO_2/Si substrate ($R_{Si/SiO2}$). Optical contrast is calculated as $C = (R_{CTPR} - R_{Si/SiO2})/(R_{CTPR} + R_{Si/SiO2})$.

S3. Contrast simulation for CTPR films on native oxide silicon substrates

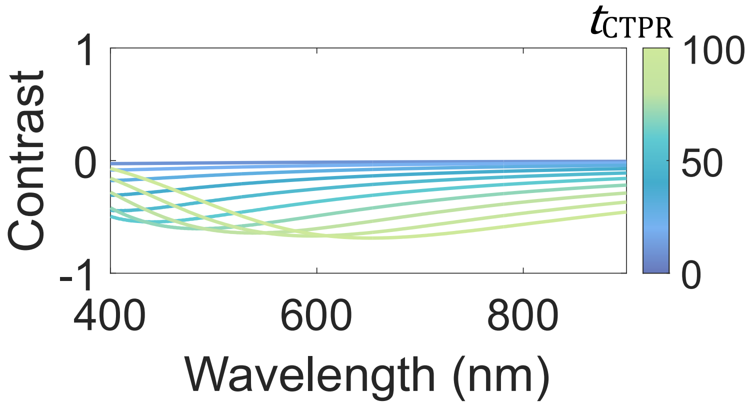

Figure S4. Optical contrast simulation for CTPR films of different thickness (t_{CTPR}) deposited on a silicon substrate with a native oxide layer with thickness of 5 nm.

S4. Optical images for spin-coated CTPR4 films at different concentrations

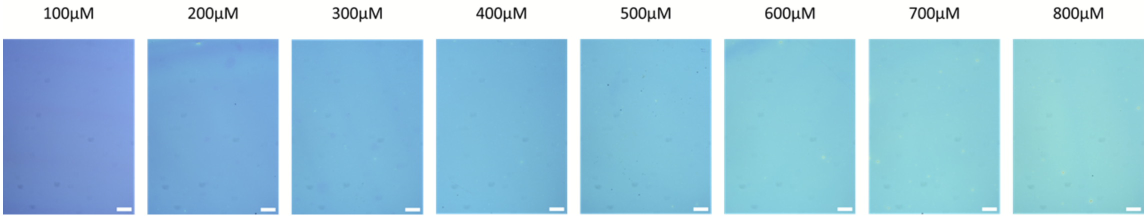

Figure S5. Optical images of spin coating CTPR4 films prepared from solutions with increasing protein concentration. Scale bar: 100 µm.

S5. Ellipsometry characterization of spin-coated CTPR samples

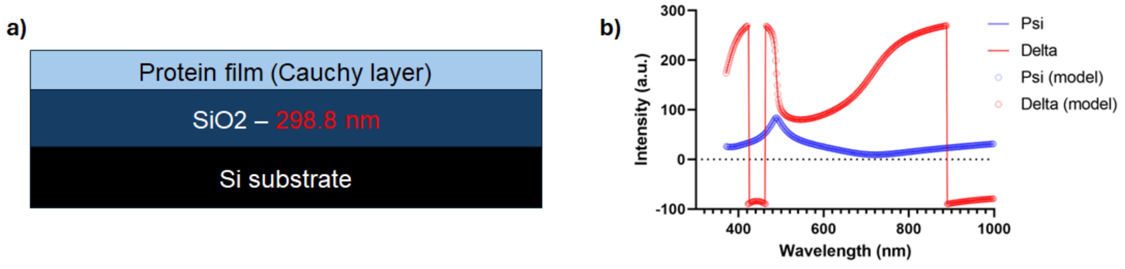

Figure S6. (a) Sketch for the Cauchy model used to fit ellipsometry spectra obtained from CTPR samples. (b) Ellipsometry spectra for a film of CTPR4 prepared by spin-coating a 400 μM protein solution. Continuous lines represent fits to the Cauchy model, from which thickness and refractive index dispersion can be obtained. Similar spectroscopy and analysis were performed for samples prepared from different concentrations. The thickness values extracted from the fits are collected in Table 1 of the main text. Refractive index values are presented in Fig. S7.

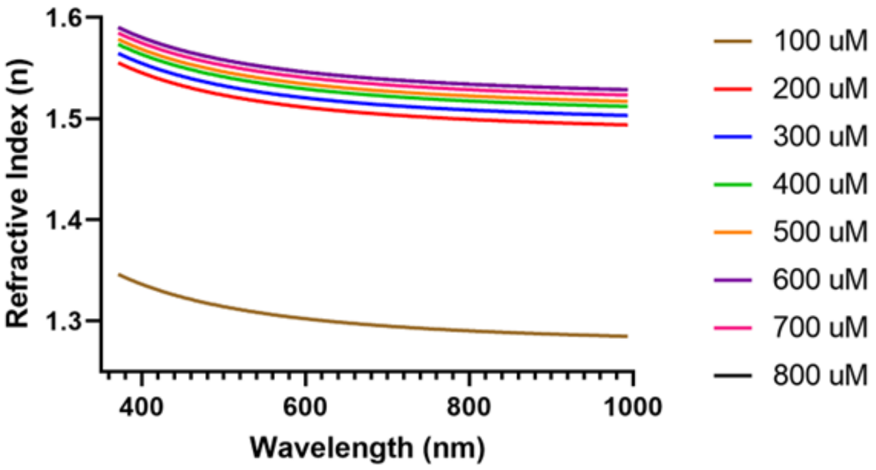

Figure S7. Refractive index values extracted from the fit to a Cauchy model of ellipsometry data for CTPR4 samples prepared from different protein concentrations.

S6. Atomic force microscopy characterization of spin-coated CTPR samples

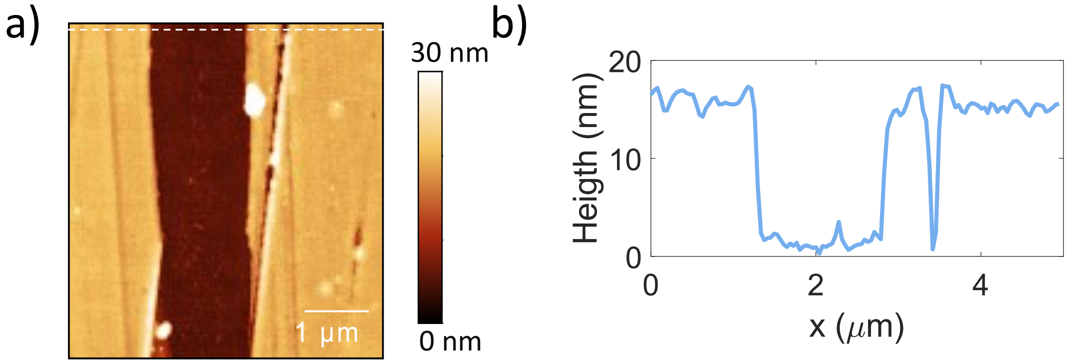

Figure S8. (a) AFM topography of a CTPR4 film, prepared by spin-coating of a 200 µM protein solution. The image is taken in a region where the film has been previously scratched with a pipette tip. (b) Height profile across the dashed line marked in panel a.

S7. Linear regression parameters

Table S1. Summary of linear regression parameters, including slopes, intercepts, and coefficients of determination for the thickness-concentration data for CTPR films presented in Figures 2 and 4 in the main text. Slope and intercept errors are obtained from the 95% confidence bounds of the fits.

**Linear regression parameters for the thickness-concentration data**						
Film Type	Technique	Sample	Slope ± Error (nm/μM)	Intercept	Coefficient of Determination (R^2)	Figure
Spin-coated	Micro-reflectance	CTPR4	0.054 ± 0.005	-1 ± 3	0.991	Fig 2a & Fig 2b
Spin-coated	Ellipsometry	CTPR4	0.050 ± 0.005	2 ± 2	0.991	Fig 2a & Fig 2b
Spin-coated	AFM	CTPR4	0.050 ± 0.005	1 ± 3	0.989	Fig 2a
Spin-coated	Micro-reflectance	CTPR8	0.075 ± 0.004	-1 ± 2	0.998	Fig 2b
Spin-coated	Micro-reflectance	CTPR16	0.23 ± 0.03	-12 ± 17	0.979	Fig 2b
Spin-coated	Ellipsometry	CTPR8	0.067 ± 0.004	4 ± 2	0.996	Fig 2b
Spin-coated	Ellipsometry	CTPR16	0.22 ± 0.03	-11 ± 15	0.983	Fig 2b
Drop-casted	Micro-reflectance	CTPR4	0.50 ± 0.03	-1 ± 5	0.994	Fig 4a
Drop-casted	Micro-reflectance	CTPR8	1.2 ± 0.1	-10 ± 14	0.993	Fig 4a
Drop-casted	Micro-reflectance	CTPR16	3.0 ± 0.3	-10 ± 40	0.991	Fig 4a

S8. Film thickness as a function of concentration for different CTPR lengths.

Table S2. Thickness values for spin-coated CTPR films obtained from micro-reflectance measurements. Three separate samples were characterized at each concentration value. Micro-reflectance spectroscopy was evaluated at five different locations for each sample. The main thickness values are obtained from the average of all the measurements. Error is calculated from the standard deviation of results.

**Micro-reflectance**			
Spin-Coating concentration (μM)	CTPR 4 thickness (nm)	CTPR 8 thickness (nm)	CTPR 16 thickness (nm)
100±3	3±2	7±2	18±2
200±6	12±2	15±3	34±2
300±8	16±2	22±2	57±5
400±10	21±2	30±2	78±5
500±10	25±2	36±2	87±2
600±20	33±2	44±2	119±3
700±20	36±2	53±2	147±2
800±20	42±2	59±2	183±4

Table S3. Thickness values for spin-coated CTPR films obtained from ellipsometry measurements. Three separate samples were characterized at each concentration value. The main thickness values are obtained from the average of all the measurements. Error is calculated from the standard deviation of results.

**Ellipsometry**			
Spin-Coating concentration (μM)	CTPR 4 thickness (nm)	CTPR 8 thickness (nm)	CTPR 16 thickness (nm)
100±3	5±1	12±3	19±2
200±6	12±1	18±2	35±1
300±8	18±1	23±1	55±4
400±10	23±1	30±1	74±5
500±10	28±1	37±1	87±1
600±20	32±1	45±1	116±2
700±20	36±1	52±2	146±1
800±20	41±1	58±3	177±4

Table S4. Thickness values for drop-casted CTPR films obtained from micro-reflectance measurements. Three separate samples were characterized at each concentration value. Micro-reflectance spectroscopy was evaluated at five different locations for each sample. The main thickness values are obtained from the average of all the measurements. Error is calculated from the standard deviation of results.

**Micro-reflectance**			
Drop-casted concentration (μM)	CTPR 4 thickness (nm)	CTPR 8 thickness (nm)	CTPR 16 thickness (nm)
5±1	3 ± 2	5 ± 2	11 ± 4
10±1	4± 2	9 ± 5	23 ± 7
25± 1	14 ± 5	22 ± 6	53 ± 11
50±1	24 ± 4	50 ± 10	120 ± 10
100±3	52 ± 7	90 ± 10	330 ± 100
150±4	70 ± 8	170 ± 10	460 ± 50
200±6	93 ± 8	220 ± 50	570 ± 50
250±7	122 ± 12	310 ± 50	690 ± 110
300±8	157 ± 14	350 ± 40	940 ± 80

S9. Thickness as a function of mass concentration for spin-coated and drop-casted CTPR samples

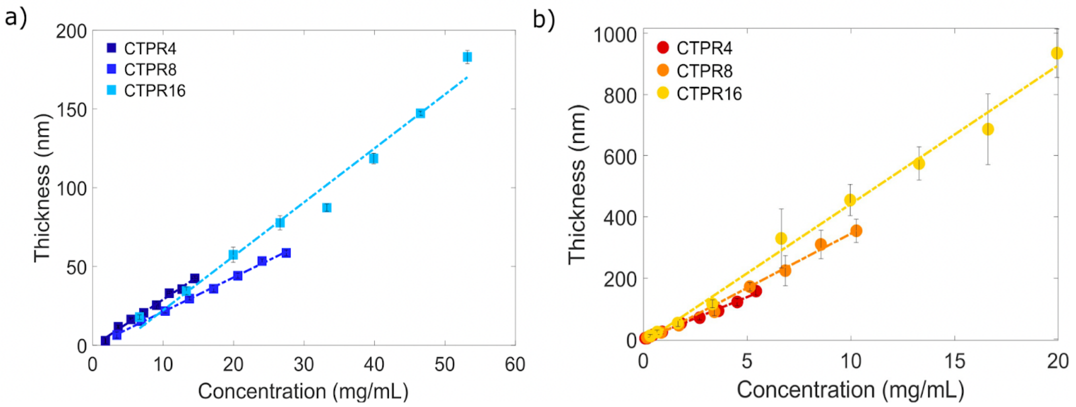

Figure S9. Thickness of CTPR films as a function of mass concentration (mg/ml) for samples prepared by (a) spin-coating and (b) drop-casting. Solid lines represent linear fits to the data.

Table S5. Slope extracted from the linear fit of thickness-concentration data in Fig. S9, for samples of different lengths prepared by spin coating and drop casting methods, respectively.

Protein	Spin-coated films Thickness-Concentration Slope ($\mathbf{nm/mg.}\boldsymbol{mL}^{-1}$)	Drop-casted films Thickness-Concentration Slope ($\mathbf{nm/mg.}\boldsymbol{mL}^{-1}$)
CTPR 4	3.0±0.3	28±2
CTPR 8	2.2±0.1	35±3
CTPR 16	3.4±0.5	45±4

As a function of mass concentration, a deviation from a simple linear correlation is observed for spin-coated films of CTPR16, for which we have prepared samples at higher concentrations. Specifically, thickness data for concentrations higher than 30 mg/mL seem to follow a linear trend with higher slope than data at lower concentrations. Interestingly, the appearance of different thickness-concentration regimes has been described for spin-coated polymer films. Schubert and Dunkel [s2] attribute these distinct regimes in polystyrene films to the variations of viscosity with concentration. The results of fitting CTPR16 thickness-concentration data to two different linear regimes with different slopes are presented in Figure S10.

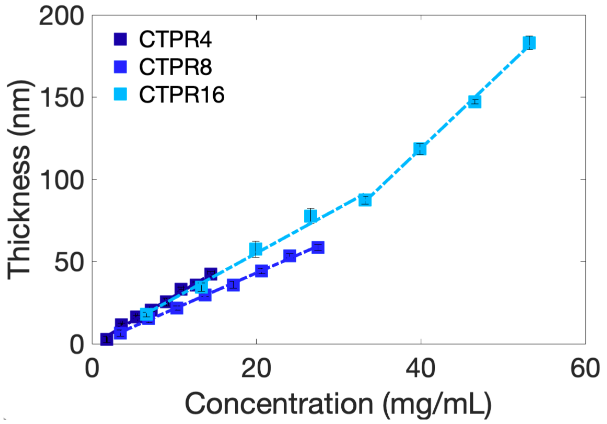

Figure S10. Thickness of CTPR films as a function of mass concentration (mg/ml) for samples prepared by spin-coating. Solid lines represent linear fits to the data. Data for CTPR16 has been fitted to two different linear expression for lower and higher concentration regimes, with corresponding slopes of 2.7 and 4.7 $\mathrm{nm/mg}.\,mL^{-1}$, respectively.

S10. Purification and characterization of CTPR4, CTPR8 and CTPR16

SDS-PAGE gel electrophoresis

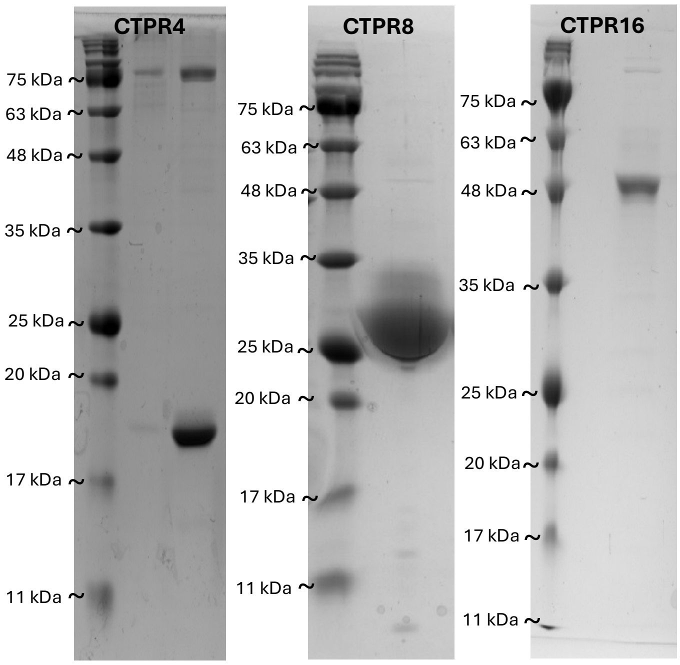

Figure S11. SDS-PAGE electrophoresis gel (15%) of purified CTPR proteins. From left to right gels corresponding to the molecular weight markers (PageRulerTM Prestained Protein Ladder) and purified CTPR4, CTPR8, and CTPR16 samples after HisTag cleavage.

Mass spectrometry by Matrix/Assisted Laser Desorption/Ionization Time-Of-Flight (MALDI-TOF)

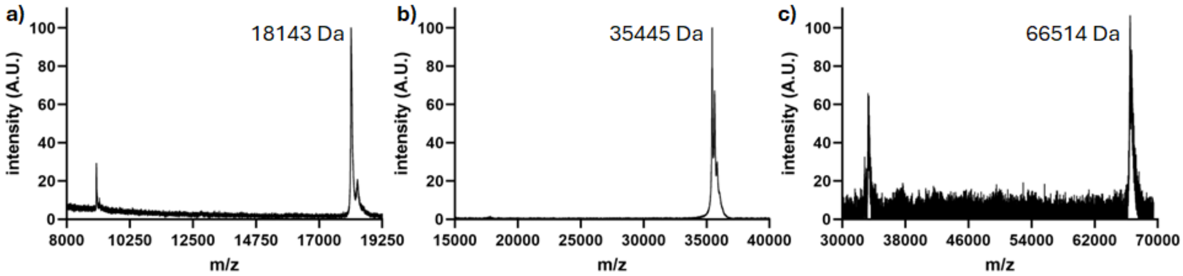

Figure S12. MALDI-TOF mass spectra of purified CTPR4 (a), CTPR8 (b), and CTPR16 (c) after His-tag cleavage. MALDI–TOF analysis was performed using a UltrafleXtreme from "Bruker". Protein samples were mixed with the matrix solution (10 mg mL^{-1} sinapinic acid, 50% acetonitrile, 0.1% trifluoroacetic acid), at a 1:3 Protein:Matrix ratio (v/v) and then spotted onto the MALDI STA µFocus plate support from "HST inc.". The experimentally calculated MWs are: 18143 Da for CTPR4, 32942 Da for CTPR8, and 66514 Da for CTPR16.

Supporting References

[s1] Byrnes SJ. Multilayer optical calculations. ArXiv preprint **2016**, arXiv:1603.02720 (2016)

[s2] Schubert, D. W.; Dunkel, T. Spin Coating from a Molecular Point of View: Its Concentration Regimes, Influence of Molar Mass and Distribution. *Materials Research Innovations* **2003**, *7* (5), 314–321.
